# Editorial for the Special Issue: Foodborne Pathogen Distribution, Ecology, Inactivation, and Methods of Differentiation

**DOI:** 10.3390/microorganisms7120701

**Published:** 2019-12-15

**Authors:** Ross C. Beier, Steven L. Foley, Roger B. Harvey

**Affiliations:** 1 Food and Feed Safety Research Unit, Southern Plains Agricultural Research Center, Agricultural Research Service, U.S. Department of Agriculture, College Station, TX 77845, USA; roger.harvey@usda.gov; 2 Division of Microbiology, National Center for Toxicological Research, U.S. Food and Drug Administration, Jefferson, AR 72079, USA; Steven.foley@fda.hhs.gov

Foodborne pathogens are a major cause of diarrheal disease throughout the world, and 40% of the foodborne illnesses are observed among children under the age of 5 years. With the overuse of antibacterial drugs, multidrug-resistant (MDR) strains have increasingly become more commonplace. Therefore, a better understanding of pathogenic bacteria and how drug resistance genes are spread is crucial in on-going efforts to control MDR pathogenic bacteria.

This Special Issue contains nine papers that contribute to aspects of foodborne pathogen ecology: genomic located resistance genes in *Salmonella* Indiana (an emerging foodborne pathogen), *Salmonella* gene expression during biofilm formation, effects of organic acids on *Campylobacter jejuni*, modulation of the immune response in poultry to reduce foodborne pathogens, spread of *Escherichia coli* from feces to lettuce, enrichment of *Listeria monocytogenes*, and methods for detection of *E. coli* O157:H7.

Genes are the basis for all phenomena observed in the bacterial world, and in this Special Issue, three papers discuss how genomics makes an impact on resistance and the ability of organisms to survive in the environment. Lu et al. [1] describe a genomic study of an MDR *Salmonella enterica* subsp. *enterica* serovar Indiana isolated from poultry at a food production site in China. This pathogen was first isolated in China in 1984 and is now an emerging human pathogen found in poultry, and is currently present in animals, processing facilities, food, and humans. This paper demonstrates that the *Salmonella* Indiana studied carries two MDR regions on its genome, which contain type I integrons and Tn*7* transposons along with several drug-resistance genes, and this genetic material may easily be transferred between organisms. This work may help improve knowledge of the spread of bacterial resistance and may help guide the management of clinically utilized drugs for the control of MDR *S.* Indiana. Shi et al. [2] evaluated differences in gene expression of *Salmonella enterica* serovars Heidelberg, Kentucky, and Enteritidis during biofilm formation. *Salmonella* can resist the effects of antimicrobials by using biofilms, and biofilms can help them thrive in a variety of ecosystems. The principle polysaccharide structural component of the biofilm is extracellular polymeric substances (EPS), which plays a main role in structural support and is regulated by the bacterial cellulose synthase (*bcs*) operon. Another major component in biofilm production is the amyloid proteinaceous curli fimbriae structures that are controlled by the curli specific gene (*csg*). The authors found that expression levels varied for the *bcsA* and *csgD* genes between the three serovars. Variation in gene expression was also observed between strains within each serovar. Data presented in these studies may lead to a better understanding of individual serovar gene expression differences during biofilm formation.

Among the top five pathogens contributing to foodborne illnesses in the U.S. resulting in hospitalizations are *Campylobacter*, *Salmonella*, and Shiga toxin-producing *E. coli* (STECs). During processing, organic acids are often used in a carcass wash to remove bacteria or may be added to poultry feed to control bacteria. Beier et al. [3] evaluated six organic acids against 96 *C. jejuni* strains isolated from broiler chicken houses at different poultry farms in several different states. The mechanism of bacterial inhibition by organic acids has traditionally been assumed to be dependent on pH or the undissociated form of the organic acids. An underlying premise of most previous studies is the organic acid must be protonated to pass through the outer bacterial membrane. The study presented here calculated both the undissociated and dissociated organic acid species at the MICs of *C. jejuni*. This study clearly shows that the dissociated organic acid levels correlate best with the molar MICs (MIC_M_s). Many of the organic acids studied are utilized as energy sources by *C. jejuni*. *C. jejuni* strains (97%–100%) were inhibited by a dissociated organic acid concentration range of 20.39–24.86 mM, except for formic and lactic acid, which are highly utilized by *C. jejuni* for energy production. Of the six organic acids studied, citric acid was the most efficient at inhibiting *C. jejuni*. Swaggerty et al. [4] reviewed the literature for immunomodulation of the poultry immune system to reduce inflammation, boost a weakened response, improve gut health, and provide an approach to prevent foodborne pathogen diseases. *Campylobacter* and *Salmonella* contamination of poultry products are leading causes of foodborne illness in the US. The authors describe that proper nutrition enhances productivity, helps maintain a healthy gastrointestinal tract and gut microflora while assisting the bird to reach its full genetic potential. The days immediately following post-hatch are critical because the birds are highly susceptible to pathogens during this time. One reason for the observed susceptibility to pathogens is the functional inefficiency of the poultry heterophils, polymorphonuclear cells (PMNs). Supplementation of the correct prebiotics can produce an immunologically superior bird by enhancing heterophil function resulting in increased resistance against *Salmonella* invasion. These studies also indicate that immunomodulation can be a means to enhance poultry health and improve food safety without adversely impacting performance. Jeamsripong et al. [5] report on the in-field transfer and survival of *E. coli* from animal feces to romaine lettuce following foliar irrigation. *E. coli* can be transferred up to 5.33 ft from feces located in a furrow near heads of lettuce by foliar irrigation. The level of *E. coli* contamination was influenced by the distance between heads of lettuce and fecal deposits, the amount of foliar irrigation, and wind aspect of lettuce with respect to the location of feces. A 5 ft no-harvest zone around fecal material can substantially reduce bacterial contamination.

The following three papers discuss important aspects of the foodborne pathogen *Listeria monocytogenes*. Davis et al. [6] reviewed the stress response literature for *L. monocytogenes* during colonization of the gastrointestinal tract of numerous hosts, including humans. *L. monocytogenes* is a Gram-positive, foodborne pathogen that can cause gastroenteritis, meningitis, encephalitis, and septicemia. The ability of *L. monocytogenes* to establish infections and colonize the gastrointestinal tract is directly related to its ability to overcome the stress factors presented by these environments. There are numerous response systems of *L. monocytogenes* that confer protection against the encountered stressors; however, the reduced oxygen availability encountered by *L. monocytogenes* has not been fully characterized. *L. monocytogenes* produces a stress response to acidity, bile, osmolarity, and temperature. When *L. monocytogenes* is studied using aerobic conditions, these conditions do not fully emulate the physiological conditions within the human gastrointestinal tract. Due to the development of new drugs that can target the bacterial sensor that determines oxygen availability, it is important that the *L. monocytogenes* stress response to oxygen deprivation be fully characterized. Parsons et al. [7] describe how lithium chloride, esculin, and ferric ammonium citrate are utilized in several enrichment schemes for *L. monocytogenes*. Many enrichment protocols for *L. monocytogenes* rely on esculin hydrolysis by *L. monocytogenes*. The authors report that inactivation of the gene, *lmo1930*, will impair the ability of *L. monocytogenes* to grow in the presence of lithium chloride and hydrolyze esculin, and as a result, reduce colony size. Their results demonstrate how *L. monocytogenes* can evade many commonly used selective enrichment protocols by inactivation of the *lmo1930* gene in the menaquinone biosynthesis operon. A paper by Olstein and Feirtag [8] describes a *Listeria* indicator broth with an improved presumptive positive performance. The original formulation of the *Listeria* indicator broth routinely exhibited false-positive test results. When d-arabitol and bromcresol purple were substituted in a modified MOX medium for esculin, field trials demonstrated that the new improved formulation significantly reduced the frequency of false-positives compared to the original *Listeria* indicator broth formulation. The new formulation resulted in no false-positive samples during field trials, while the original formulation had 54% increased presumptive positive samples.

Zhao et al. [9] discuss that false-negative results in the PCR assay can be caused by various constituents in foods. However, using an internal amplification control can measure the presence of false-negative results in the PCR assay. The authors’ results indicate that real-time fluorescence PCR combined with an internal amplification control possess the characteristics of stability, sensitivity, and specificity. The improved methods described here can potentially provide fast and sensitive detection of *E. coli* O157:H7 with accurate quantification while preventing false-negative results in contaminated samples.
